# Smart Visualization of Medical Images as a Tool in the Function of Education in Neuroradiology

**DOI:** 10.3390/diagnostics12123208

**Published:** 2022-12-17

**Authors:** Aleksandar Simović, Maja Lutovac-Banduka, Snežana Lekić, Valentin Kuleto

**Affiliations:** 1 Department of Information Technology, Information Technology School ITS, 11000 Belgrade, Serbia; 2 Department of RT-RK Institute, RT-RK for Computer Based Systems, 21000 Novi Sad, Serbia; 3 Department of Emergency Neuroradiology, University Clinical Centre of Serbia UKCS, 11000 Belgrade, Serbia

**Keywords:** smart visualization, medical imaging, SVMI, neuroradiology, education

## Abstract

The smart visualization of medical images (SVMI) model is based on multi-detector computed tomography (MDCT) data sets and can provide a clearer view of changes in the brain, such as tumors (expansive changes), bleeding, and ischemia on native imaging (i.e., a non-contrast MDCT scan). The new SVMI method provides a more precise representation of the brain image by hiding pixels that are not carrying information and rescaling and coloring the range of pixels essential for detecting and visualizing the disease. In addition, SVMI can be used to avoid the additional exposure of patients to ionizing radiation, which can lead to the occurrence of allergic reactions due to the contrast media administration. Results of the SVMI model were compared with the final diagnosis of the disease after additional diagnostics and confirmation by neuroradiologists, who are highly trained physicians with many years of experience. The application of the realized and presented SVMI model can optimize the engagement of material, medical, and human resources and has the potential for general application in medical training, education, and clinical research.

## 1. Introduction

Over the last decade, imaging systems and devices have undergone significant advances in medical research, such as the use of magnetic resonance imaging (MRI) [1], multi-detector computed tomography (MDCT) [2], and positron emission tomography (PET) [3]. In the last seven years, the application of new technologies such as machine learning (ML) and artificial intelligence (AI), driven by the availability of large data sets (i.e., Big Data), has made astonishing progress in the ability of machines to process and manage data including images [4], language [5], and speech [6]. Furthermore, the rapid development of computer science in collaboration with medicine can contribute to developing new algorithms for disease prediction in medicine and new methods of medical image processing. The healthcare sector can significantly benefit from the application of these new information technologies and the exponential growth of the total generated data (150 EB or 10^18^ bytes in the United States alone, growing 48% annually [7,8]), as well as from the new approaches in designing a medical information system.

In August 2018, the National Institutes of Health discussed gaps in medical imaging, defining key research priorities including automated imaging methods, extracting valid information from medical imaging, electronic phenotyping, and prospectively structured medical imaging reporting [9]. In addition, medical diagnostics can be improved using modern methods of visualizing large amounts of medical images, which can sometimes be difficult to classify by human experts and specialists. Additionally, AI involving ML and Big Data has the potential to improve clinical outcomes and further increase the value of medical images in a way yet to be defined. It also remains a condition for achieving performance at the physician level [10,11,12]. However, the uncertainty and unreliability of the results obtained by Big Data analysis may result in the fall of perspective models [13]. Despite the evident progress of AI applications in medical imaging and the significant financial investments of large global companies in developing new technologies, radiologists are slowly adopting AI in their work. In a study conducted in the United States in 2020, 90% of 50 radiologists surveyed felt that the enhancement of imaging quality through AI would become mainstream in less than five years [14]. Still, although the advancement in information technology and medical image processing devices is growing exponentially with the synergy of human knowledge, physicians, and researchers, the benefits of computer-assisted intervention and diagnosis are viable.

Many researchers propose different approaches to analyzing medical images using available technologies and automatic processing systems. In the past few years, the analysis of medical images based on ML has gained significant importance in scientific research. In particular, with the progress of computer vision, researchers are encouraged to develop various systems for the analysis, correlation, and interpretation of medical images [15,16,17] such as convolutional neural networks for brain image segmentation [18,19,20,21]; for brain tumor detection and classification [22,23,24]; medical image registration, fusion, and annotation [25,26,27,28,29]; computer-aided diagnosis (CAD) systems [30,31,32,33,34,35]; and the automatic detection of micro-bleeds in a medical image [36,37,38].

This paper presents a new SVMI method for visualizing medical images with a new approach to medical image processing in neuroradiology and a more precise insight into patient brain changes by rescaling and coloring pixels of significance for displaying the disease. The motivation behind this study is the need for a comprehensive survey in the field of medical imaging visualization that approaches the issue from the educational perspective as a foundation of knowledge creation and dissemination in society.

The paper is organized as follows. First, it reviews the literature on different techniques and approaches to medical image processing. In Section 2, we present the standard MDCT diagnostic method in neuroradiology, after which we present our smart visualization (SVMI) method in detail through the detailed description of medical image processing. In Section 3, we give a technological description of the developed SVMI model, followed by an evaluation of the system and results. The following section is a discussion that includes the limitations and theoretical and practical implications of this research. Finally, we give a final opinion on the subject matter and outline future work.

## 2. Materials and Methods

### 2.1. Standard Methods and Existing Problems

**Figure 1:**
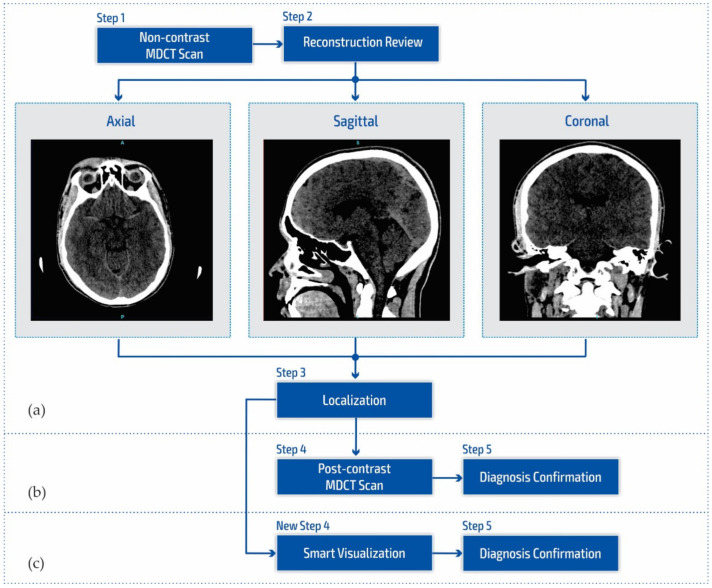
Block diagram of (**a**) the multi-detector computed tomography method, (**b**) the post-contrast MDCT diagnostics method, and (**c**) the smart visualization (SVMI) method.

Figure 1a shows a block diagram of the standard neuroradiology diagnostics approach using a multi-detector computed tomography. Following MDCT diagnostics and a non-contrast MDCT endocranium scanning (Figure 1a, Step 1), a neuroradiologist examines the reconstruction in all three cross-section planes (Figure 1a, Step 2): (a) axial, (b) coronal, and (c) sagittal to determine the existence of pathological changes in density, classified as hyperdensity [39]; hypodensity [40]; and isodensity [41], to accurately determine the localization of the region of interest (Figure 1a, Step 3).

After determining the existence of a pathological change, the work of a neuroradiologist requires access to post-contrast MDCT diagnostics (Figure 1b, Step 4) to characterize the changes in the brain and develop a conclusion in the form of differential diagnosis and medical determination (Figure 1b, Step 5). Unfortunately, rescanning exposes the patient to the possible consequences of an additional influence of ionizing radiation due to its cumulative effect. Side effects can also be reflected in the patient’s allergic reactions to iodine that have not been previously determined. This includes the inability to use iodine contrast media in pre-existing and confirmed allergic reactions, which consequently refers the patient to MRI diagnostics that are not available to everyone because they have both absolute and relative contraindications. With the goal of successful treatment and diagnosis of brain diseases in patients, current research in MDCT and MRI diagnostics aims to characterize brain diseases non-invasively [42,43,44,45,46]. However, multi-detector computed tomography has some advantages concerning MRI diagnostics, enabling quantitative measures of deterioration of the blood–brain barrier that may be associated with tumor and neovascularization [42,43,47,48].

Due to the stated conditions and limitations in Figure 1b at Step 4, in order to prevent post-contrast scanning of the patient, a New Step 4 in Figure 1c is proposed which enables the smart visualization of medical images (SVMI) and a more precise presentation to neurologists of the localized changes in the patient’s brain at a determined cross-section of plane and slice.

### 2.2. Smart Visualization Method (SVMI)

**Figure 2:**
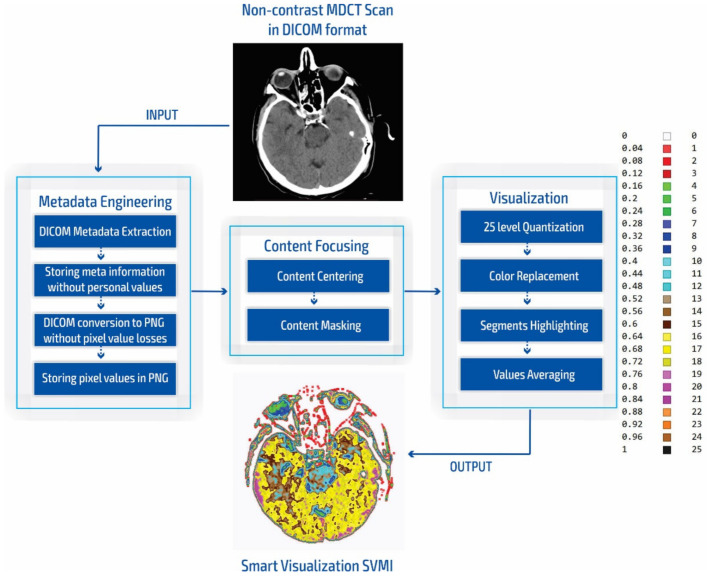
SVMI model flow chart.

The smart visualization method in Figure 1c is shown through the developed SVMI-model flow diagram in Figure 2 and explained below.

During our experiment, we compared the simulation of standard practices of neuroradiologists for the placement of grayscale images to measure the Hounsfield units with our smart visualization model. Our developed SVMI model employs a 16-bit total-pixel image without loss in conversion and without personal metadata from the DICOM format, ensuring complete protection of patients’ identities. Radiographs do not have absolute pixel values, while MDCT images can be quantified in the absolute values of the Hounsfield unit used for comparison [11]. Furthermore, our masking of medical images allows for the re-scaling of pixel regions in the brain where the neuroradiologist locates a possible change in unenhanced imaging. Accurate localization is essential for understanding the results of medical imaging and urgent cases, including the quick confirmation of the reliability of the diagnosis for a possible surgical approach. Some previous studies that also proposed algorithms that provide maps of regions of interest [10,12,49,50] did not confirm the accuracy of localization compared to neuroradiologists [10,12,50,51] or did not show reliable accuracy [49].

**Figure 3:**
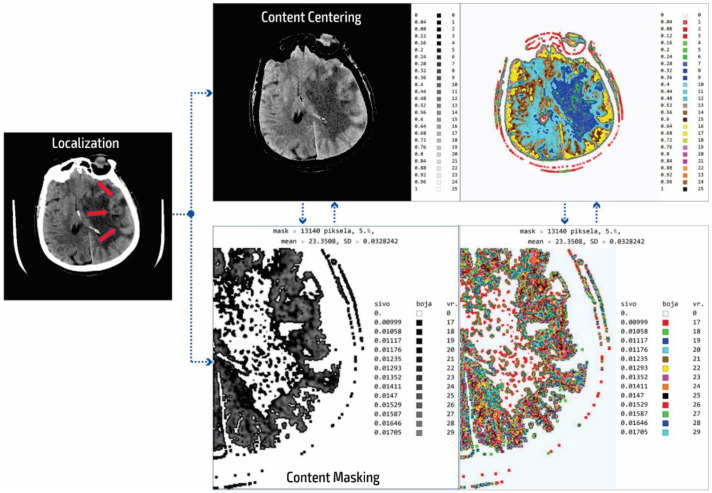
Summary of the system outputs.

In our research approach, in marking content and regions of interest all values that do not contribute to the content are set to a pixel value of zero. In this way, the levels of black (which have a value of zero or slightly greater than zero), white values (which have a maximum value), and values of gray that are not contained in the central part of the gray are set to zero so as not to interfere with the physician conclusion while observing the central segment. At the same time, white parts (for example, bones) do not affect the mean value of the observed part. The smart visualization and the summary of the system output of the developed SVMI model is shown in Figure 3.

After determining the localization of the appropriate level of a particular cross-section by an experienced neuroradiologist, centering and masking of contents and regions of interest are performed. For a non-contrast MDCT scan in a DICOM-standardized format file, in which integers represent all pixels, it is necessary to extract useful information. Therefore, only the gray range is observed so that all gray values are less than a particular value, all values greater than the other selected value are observed, and all are set to zero so as not to affect the mean value of the selected segment. Adjacent pixel values are colorized differently, meaning that physicians do not need to recognize close levels of gray because segments that have similar pixel values are different. By masking this content, a specific part can be selected (above, below, left and right 1/3, or 2/3 vertically and horizontally) so that only the part of interest is magnified. On the right, relative values of gray from zero to one are provided with a label that shows the color. The minimum and maximum values are chosen arbitrarily so that only the range of gray is observed. All other values are zero and do not affect the mean and standard deviation. Assigned colors can be changed according to the physician’s preferences to provide a clearer view of image segments with the same pixel value. The mean and variance are calculated for the selected range. It is also possible to perform averaging so that the close ranges are more clearly separated from the rest of the pixels.

The disadvantage of existing methods is that the images may show pixels that do not contain valuable information, making it necessary to identify those areas of interest and hide unnecessary pixels. In analyzing many images from the selected device, it was determined that the images contained about 80 different integer values and that the valuable part of the information was presented with 25 integer values. As the gray levels in the original image are normalized from zero to one, which is 30% useful in the display of the gray image used by the physicians, a scaled image with values from zero to one should be rescaled. This achieves 25 adjoining integer values displayed with all 100% in the range from zero to one. The algorithm first determines the central part of the image containing helpful information about soft tissue and eliminates pixels formed outside of the central part perimeter. Following this, the smallest and largest integer pixel values representing soft tissue are determined and all other pixel values are eliminated. Next, the part of the soft tissue where an anomaly can be expected is selected. Only the useful part with values from 1 to 25 are displayed as gray levels ranging from zero to one. In the content masking stage, all other parts of the background, bones, and the darker parts of anomalies that are not in the focus of interest are presented in white. The physician can choose several gray levels less than 25 to eliminate all other pixels that are not in the focus of interest. In the displayed image (Figure 3) of the content masking stage, the gray levels from 0.00999 to 0.01705 are selected as part of the range from zero to one, represented by the integer values of the original image from 17 to 29. The disadvantages of the usual approach to the analysis are that the gray levels are only 30% of the range from zero to one and that pixels may be used that significantly increase the error in the display and statistical data because, for example, on the localized part of changes in the patient’s brain where it is possible to not expect an anomaly there are also darker parts as well as bones and other side effects that do not reflect the soft tissue. Gray and colored pixel levels can be defined according to the selected range to show the color difference of adjacent levels. Additionally, it is sometimes necessary to perform additional processing to, for example, average the pixel values with neighboring values.

It is important to note that this processing does not change the image content and that all pixel values correspond to the original image. Image processing achieves better visibility where the gray levels from the original image are located. The mean value is 23.35 for soft tissue pixels without damaged parts and bone, so the statistical data (mean value and deviation) are only shown for the colored or gray parts of the images.

## 3. Results

### 3.1. Technological Description

**Figure 4:**
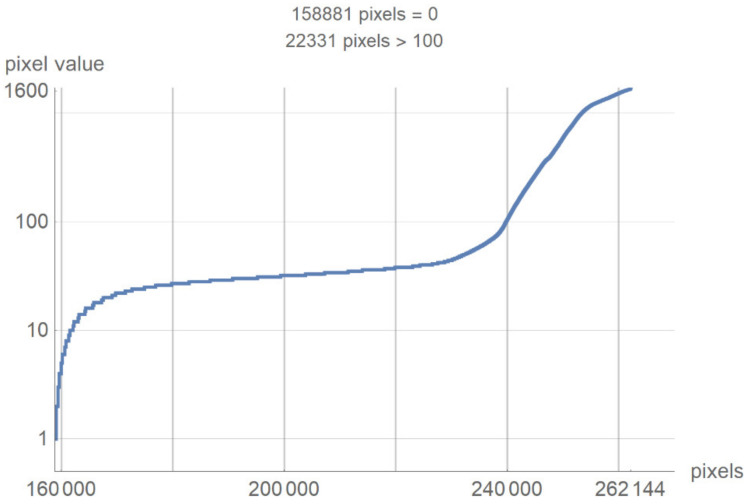
The number of pixels as a function of value.

This chapter presents a technological description of the developed SVMI model, which describes image processing according to the proposed method. The image used in the survey questionnaire to evaluate the developed SVMI model was selected for its illustration and technological description. The primary characteristics of the selected image are that it has 512 × 512 pixels or a total of 262,144 pixels of integer-type. Pixel values remain unchanged when the image format converts. The analyzed image in PNG format has the same pixel values as the original image, so the image has no losses after processing. However, many pixels do not contain valuable information, and the first task of the software is to eliminate all pixels from the image that may be misinterpreted or confusing. For the analyzed image, for example, the number of pixels with zero values were calculated. Out of 262,144 pixels, as many as 158,881 pixels had a value of zero. Useful pixels with small values (for example, 1, 2, 3 ...) are difficult to distinguish from values of zero, so it was necessary to eliminate these pixels before analysis by an expert. The number of pixels as a function of value is shown in Figure 4.

**Figure 5:**
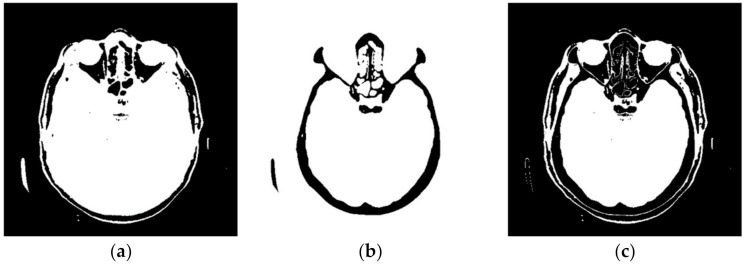
Pixels that do not contain useful information: (**a**) black represents the pixels that have a value of 0; (**b**) black represents the pixels that have a value > 100; and (**c**) black represents the pixels that have a value of 0 and a value > 100.

Figure 4 shows a large number of pixels with values between 10 and 100 and 22,331 pixels have a value greater than 100, while the most considerable value of pixels is 1682. Some programs can automatically adjust the image display to highlight pixels that improve the display of a portion of the image. For example, to increase the contrast or brightness of a portion of an image with a large number of pixels., image adjustment can be used to adjust pixel values so that more of the image content is in the visible range or to correct lousy illumination or contrast. The purpose of this software is not to distort the image display without a specialist’s control, but to accurately display the part of the image that contains valuable information on a linear scale and remove pixels from the image that do not valuably contribute to the image content. Pixels with an immense value make it impossible to display valid pixels on a linear scale from zero to one and increase the average value of helpful information pixels. One way to remove un-useful pixels from an image (Figure 5a) is to set high-value pixels to zero (Figure 5b) and to generate a pixel mask, shown in black in Figure 5c.

**Figure 6:**
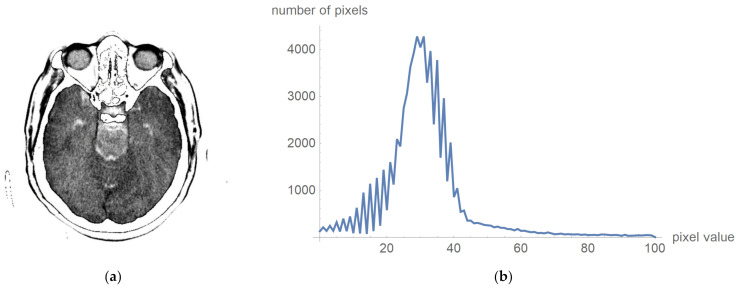
Original image multiplied by the mask and its histogram: (**a**) the negative of the original image multiplied by the mask; and (**b**)its histogram, whose pixels are higher than 0 and less than 100.

To prove that valuable information is not lost, Figure 6 shows the original image multiplied by the mask and its histogram according to Figure 5c. The negative is presented to clarify that this procedure does not damage valuable information. Figure 6a presents that the central part does not contain high-value pixels.

One can now look at what a histogram of pixel values greater than zero and less than one hundred looks like, as is shown in Figure 6b, which demonstrates what the display range for visual analysis by a specialist can be for an image whose values will be in the range from 5 to 55, i.e., that the upper threshold for the pixel value does not need to be 100. To more clearly identify the pixel values, we have chosen to display 24 different levels in which the expert can choose which range they want to observe. All other values are associated with a mask that appears to be zero, and these values are not counted as the mean value.

**Figure 7:**
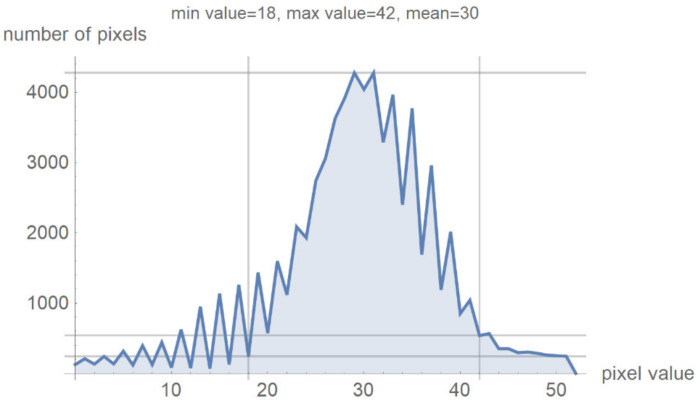
Image histogram for an arbitrarily selected range of up to 24 successive pixel values.

Figure 7 shows a histogram for 24 successive levels from the range {18, 42}, for which a mean value of 30 was calculated. Based on the histogram in Figure 7, the specialist can select the range of pixel values they wish to observe by masking all other values by setting them to 0.

**Figure 8:**
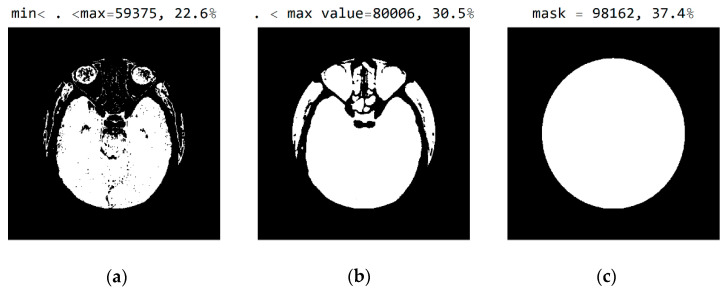
White represents pixels that will display the correct values: (**a**) in the range from minimum to the maximum selected value; (**b**) in the range of values from 1 to 55; and (**c**) in the central part of the image for the selected mask.

In Figure 8, it can be seen that the third image (Figure 8c), comprising the central part of the circular image, contains 37.4% of the total number of pixels; that the second image (Figure 8b), comprising values from the pixel range from 1 to 55, contains 30.5% of the total number of pixels; and that the mask (Figure 8a) for the selected range of 24 successive pixel values contains 22.6% of the total number of pixels. By choosing the range of pixel values, the specialist can more easily identify the pixel values of interest to conclude the type of disease.

**Figure 9:**
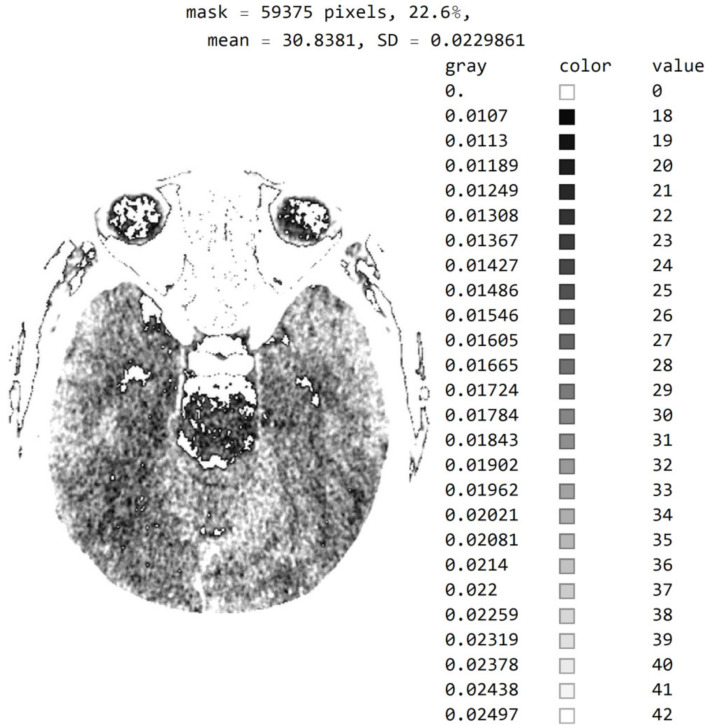
Image with exact integer values with shades of gray scaled from black (pixel value 18) to white (pixel value 42), with all other pixels outside this range shown as 0, i.e., white on the image.

Figure 9 shows an image with exact integer values with shades of gray scaled from black (pixel value 18) to white (pixel value 42), with all other pixels outside this range shown as 0 (white in the picture). The far-right column contains the exact pixel values where it can be observed that the differences of adjacent values are scaled from 0 to a maximum of 1682 on the gray range from 0 to 1, approximately 1/1682 ≈ 0.0006, which makes it impossible to accurately determine the differential values in the original image without using algorithms to highlight a range of values. In addition to displaying pixels whose values are 18 to 42, image rescaling was performed, eliminating the display of parts of the image outside the mask of the circular segment that do not include valuable information. Since the specialist chooses the range of pixel values, Figure 9 clearly shows white parts that are not displayed but could contain valuable information. The specialist may choose a different range if they wish to see pixels from the pixel range of 1 to 55 and consider those pixels to be useful in diagnosing the disease.

**Figure 10:**
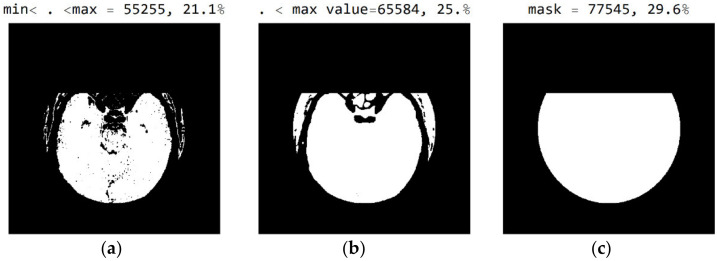
White represent pixels that will display the correct values for the selected mask: (**a**) in the range from the minimum to the maximum selected value; (**b**) in the range of values from 1 to 55; and (**c**) in the part of the image for a selected mask.

The three Figure 10a–c show black pixel masks that will set all image values to zero so that white pixels can be displayed in the image. This mask has an additional setting of zero for all pixels in the upper third (near the eyes). The new mask allows for the display of 29.6% of the total number of pixels (Figure 10c); the mask with values outside the pixel range from 1 to 55 contains 25% of the pixels (Figure 10b); and the selected range of display contains 21.1% of the total number of pixels (Figure 10a).

By choosing the range of pixel values, the specialist can more easily identify the pixel values of interest to conclude what type of disease is presented.

**Figure A1:**
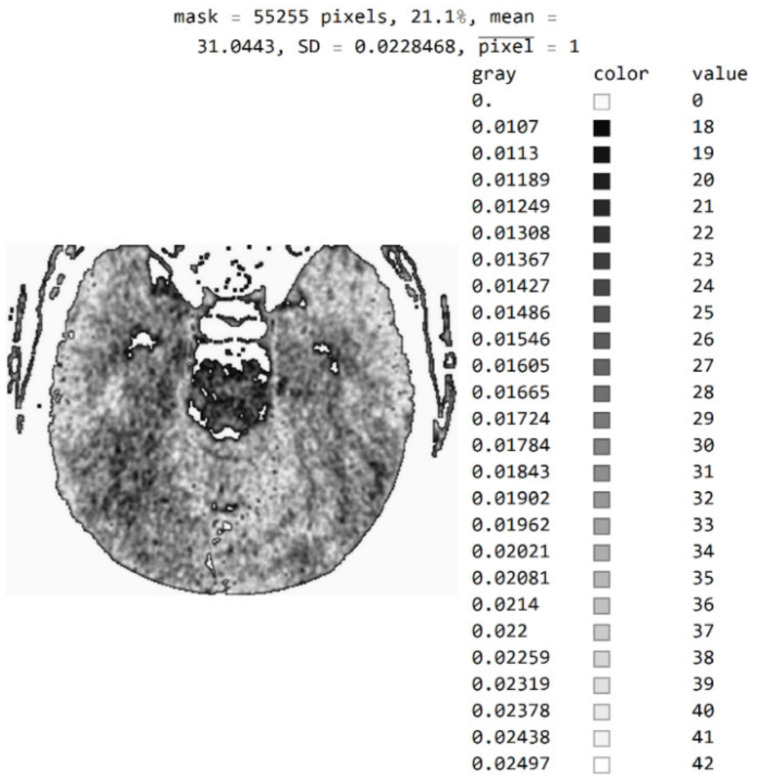
Image with exact integer values, the shades of gray scaled from black (pixel value 18) to white (pixel value 42), with all other pixels outside this range displayed as 0 and shown in white.

Figure A1 in Appendix A shows an image with exact values of integer type, shades of gray scaled from black (pixel value 18) to white (pixel value 42), and with all other pixels outside this range shown as zero, i.e., white. The description is the same as in Figure 9. By choosing a mask, the specialist may choose not to show the part of the picture that, in their opinion, does not contribute to the conclusion about the type of disease.

**Figure 11:**
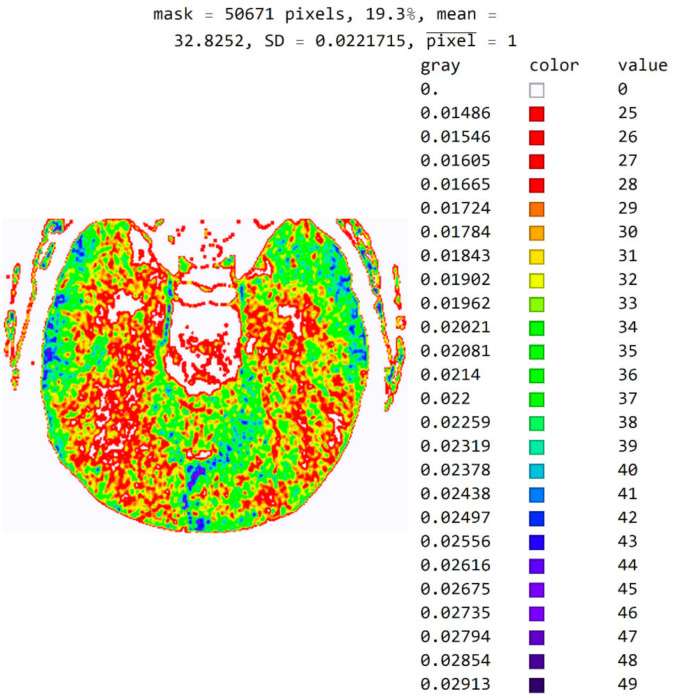
Image with the exact values of an integer type on the color map of the visible spectrum from pixel values 25 to 49. All other pixels outside this range have a value of 0 and are displayed in white.

The software also allows pixels to be displayed in color to help identify pixel values in an image. For example, in Figure 11, a color map based on light whose wavelength is in the most commonly used range in radiology (i.e., colors based on light wavelength in nanometers, Physics-Oriented Color Schemes) was selected. The color map may be different depending on what the specialist wants to emphasize to differentiate the values of adjacent pixels. In Figure 11, the minimum value was moved to 25 and not 18 because the specialist expected to see where the pixel values greater than 25 are. It is noted that a small number of pixels have a value greater than 40 and that they do not contribute to the recognition of the disease. Therefore, the specialist can choose a narrower range of values to display. High resolution between adjacent levels can negatively affect disease recognition, so it is desirable to perform averaging so that each pixel receives the average of several adjacent pixels in the image.

**Figure 12:**
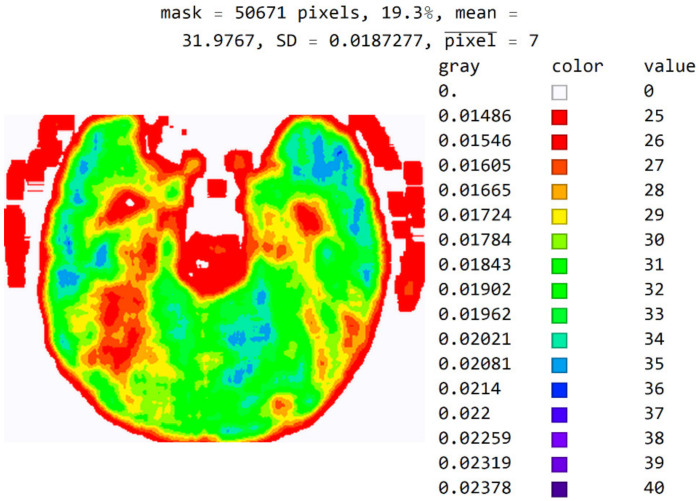
An image display with averaged seven adjacent pixels on a color map of the visible spectrum from pixel values 25 to 40. All other pixels outside this range have a value of 0 and are displayed in white.

In Figure 12, the mean value of seven adjacent pixels was calculated and averaged from 25 to 50 pixels for the display range. Figure 12 was used in an online questionnaire to evaluate the developed SVMI model. The results showed that, in the practical education of MDs in the radiology residency training program, a decision on diagnosis was made in which 95% of the surveyed physicians made the correct diagnosis after SVMI processing and this diagnosis was confirmed followings additional diagnostics.

In Figure 11 and Figure 12, all masked pixels are shown in white.

The purpose of this software is not to use just one image in the training phase and education. Instead, specialists should experiment with a wide range of values for observation, the choice of the color map, and the choice of the number of adjacent pixels for averaging to gain better insight into the areas that can contribute to the correct medical decision. 

**Figure A2:**
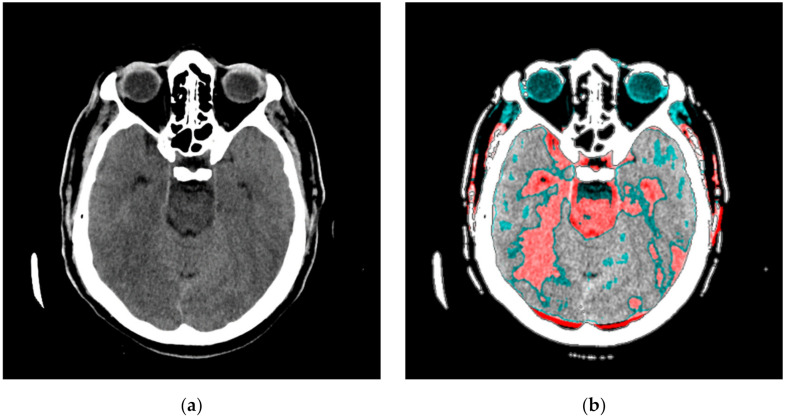
An image in gray levels with the averaged red part from processing. (**a**) An image display in gray levels and (**b**) an image display with the red, averaged part added.

Figure A2 in Appendix A shows the image in gray levels with an added red, averaged part in which the image processed with shades of gray can be combined with the colored image so that the image in gray levels has an added segment marked with red. This segment is obtained by processing and averaging.

**Figure A3:**
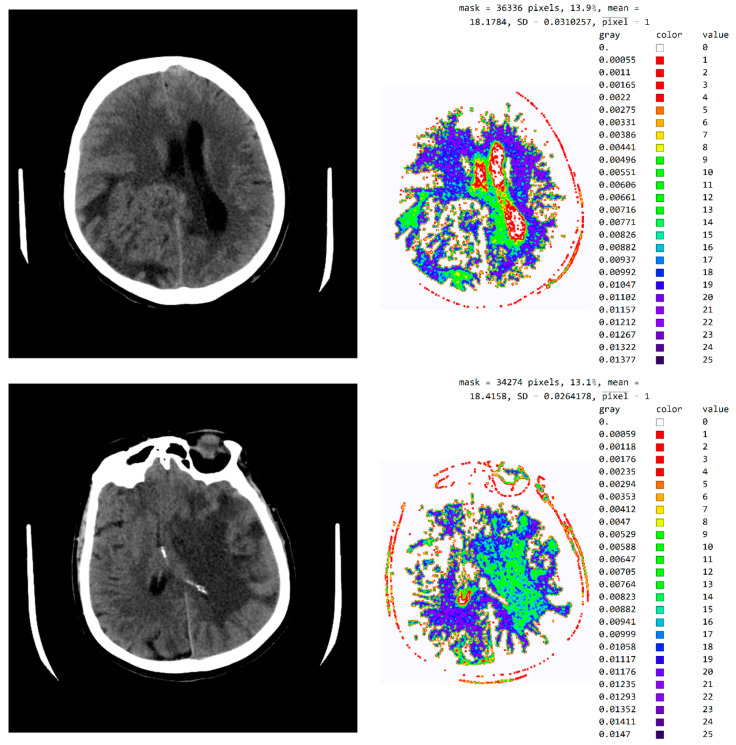

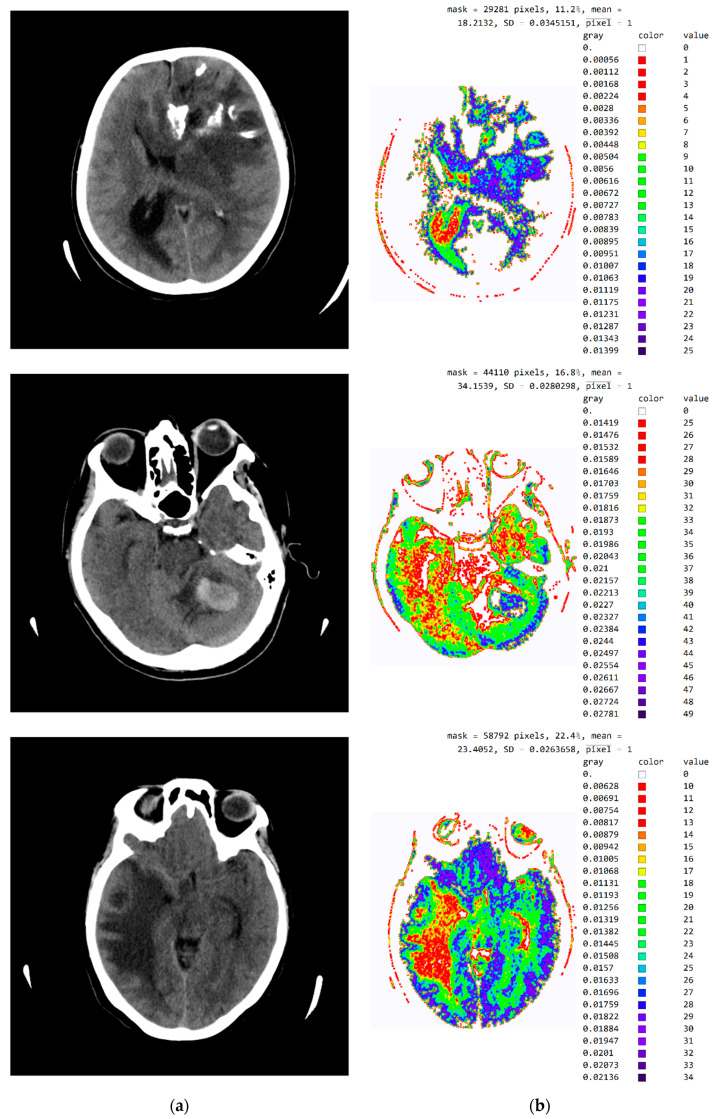
SVMI processing of non-contrast MDCT image. (**a**) Non-contrast MDCT image and (**b**) Smart Visualization of Medical Images SVMI.

Figure A3 in Appendix A provides a tabular presentation of the various ranges of values and color maps in SVMI processing for different diagnosed brain diseases.

Due to the need for the specialist to select the pixel range for display, color map, and the number of pixels for averaging, this analysis requires an interactive analysis of the specialist when selecting parameters. It cannot be used for automatic analysis and conclusion of the disease.

### 3.2. Evaluation of the Developed SVMI Model

**Figure 13:**
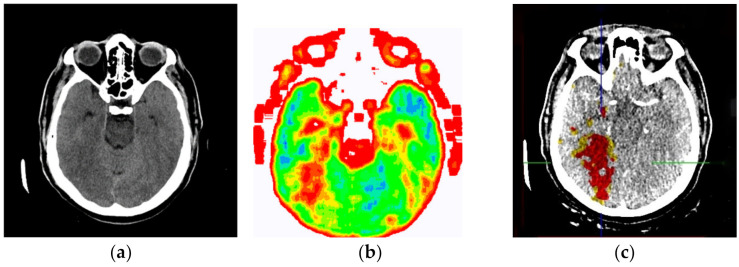
(**a**) Non-contrast MDCT image of the endocranium; (**b**) SVMI of the non-contrast MDCT image of the endocranium; and (**c**) MDCT perfusion of the same cross-section.

The evaluation of the developed model was conducted through an online questionnaire to determine whether smart visualization of medical images (SVMI) can contribute to the education of MDs in the radiology residency training program and undergraduate medical students. A total of twenty Physicians of the Department of Emergency Neuroradiology expressed their professional medical opinion when completing the questionnaire. Nine were neuroradiology/radiology specialists (45%) and eleven were MDs in the radiology residency training program (55%). All physicians agreed to participate in the research and did not have insight into the patient’s final diagnosis (Diagnosis of the disease after performing all the diagnostic methods provided by the protocol: Occipital right, in the region of PCA vascularization, observed zone of hypodensity, without differentiation of gray-white mass and flattened sulci diff.dg.acute ischemic stroke) after complete diagnostic methods, which included a post-contrast MDCT of endocranium, MDCT perfusion, and MDCT angiography. Therefore, a triple-blind review system was applied. No participants knew the physician’s opinion (before or after), or the patient’s official diagnosis, name, and surname, or the neuroradiologist who performed the examination. Additionally, all survey participants were asked questions according to the order of appearance of the images, starting with Figure 13a.

Figure 13a shows a non-contrast MDCT image of the endocranium establishing a zone of hypodensity without clear differentiation of the gray–white mass on the occipital right, which corresponds to an acute ischemic lesion. Figure 13b presents our method of smart visualization of the medical images (SVMI) of the non-contrast MDCT image of the endocranium of the same cross-section. Figure 13c, after additional diagnostics of MDCT perfusion, shows a perfusion deficit that can be seen in the same region—occipital right—which confirms the diagnosis of an acute ischemic stroke.

On the presented non-contrast MDCT image of the patient’s endocranium (Figure 13a), 100% (nine) of radiology specialists noticed that changes in the brain had occurred based on their knowledge in the field of neuroradiology, which ranged from 3 to 15 years of experience for all radiologists who expressed their expert opinion. On the other hand, based on the non-contrast MDCT image of the endocranium (Figure 13a), 81.81% (nine) of the total number of MDs in the radiology residency training program who participated in the questionnaire did not notice the change, did not adequately characterize it, or did not give an adequate differential diagnosis. Table 1 shows the analysis of the physicians’ answers to the first question observing only Figure 13a.

Following smart visualization (SVMI) of the non-contrast MDCT image of the endocranium (Figure 13b), out of all physicians whose opinions were requested, 95% of respondents made an accurate diagnosis and confirmed the existence of pathological changes in density. However, one MD in a radiology residency training program answered incorrectly. Table 2 shows the analysis of the physicians’ answers to the second question observing only Figure 13b.

The certainty of physicians in their statements, observing only Figure 13b, was performed using the five-point Likert scale. Table 3 shows the analysis of the answers given to the third question.

Physicians gave their opinion, presented in Table 4 as to whether this smart visualization (SVMI) can contribute to the education of MDs in the radiology residency training program and undergraduate medical students.

An analysis of the responses, in which 60% of physicians gave a statement that “it can” and 35% of physicians said that “to some extent it can,” argues that smart visualization of medical images (SVMI) can contribute to education. Additionally, the proposed SVMI model follows the clinical protocols for further diagnostics and patient treatment. For confirmation, Figure 13c shows MDCT perfusion in which a perfusion deficit is observed, articulating in favor of the existence of an acute ischemic stroke.

## 4. Discussion

Given their knowledge and many years of experience in the field, when neuroradiologists notice a change in the non-contrast image, they may have a certain kind of sureness about the type of change in the brain. However, applying an IV (intravenous) contrast agent is necessary to characterize the visualized pathological change in density. From the existing medical documentation, neuroradiologists can understand what kind of change is being observed from previous examinations of non-contrast and post-contrast images. Therefore, it is not necessary to apply contrast media for characterization, but only in the form of disease progression, relapse, or recurrence (in post-operative conditions, after chemotherapy or radiation therapy). If the physician’s specialist has information in the form of a report on the type of pathological change in density, and when there is no need for post-contrast imaging (inpatient disease), the use of SVMI to educate MDs in the radiology residency training program as well as undergraduate medical students becomes important and gains high significance. 

Giving an IV contrast agent is necessary when it is difficult to notice changes because the pathological change from applying contrast opacified characteristically. Pathological changes in density at the sub-centimeter level do not have to show indirect signs of existence on the non-contrast MDCT scan. If there is prior knowledge about the malignant disease it is necessary to provide a contrast to determine the endocranial dissemination of the underlying condition. In patients with existing changes in the brain parenchyma by type of secondary deposits and new neurological outbursts, re-administration of contrast medium to the patient can be avoided and the proposed SVMI model could be applied to determine possible newly formed changes of the same type.

The weak point of the proposed method is the lack of automated diagnosis. Therefore, future improvements will include (1) conducting deep learning techniques with a massive dataset already used by experts to train novices and (2) the development of a graphical user interface (GUI) to simplify the expert’s interaction with the software in a way to get the desired response.

## 5. Conclusions

This paper presents a novel model of smart visualization of medical images (SVMI) obtained through MDCT diagnostics and an innovative method for their reconstruction and improvement, ensuring that the regenerated medical image of the human brain is suitable both for interpretation and as an accompanying tool in diagnostics in radiology residency training programs as well as for education for medical students’ undergraduate studies. With the proposed model of smart visualization of medical images (SVMI), the domain of neuroradiology can experience multiple benefits from the aspect of education. Moreover, a high-performance model of innovative image visualization of specific cross-sections of interest mimics the workflow of neuroradiologists.

Radiology specialists with many years of experience as well as MDs in the radiology residency training program have confirmed that the proposed model could make a significant contribution both in the radiology residency training program and in a more accurate visualization of the region of interest where changes in the brain occur. Of the surveyed physicians, 95% made a correct diagnosis after SVMI processing and confirmed that it could contribute to education in neuroradiology, providing reliable ground truth. In addition, to our knowledge, the method of smart visualization of medical images at the cross-section level for the purpose of educating future radiologists has not been previously tested in scientific research.

An equilibrium between the complexity and quality of medical data obtained through MDCT diagnostics and the careful visualization of images with seclusion segments of interest that can be adapted to all medical applications in healthcare institutions is an essential segment for the future development of smart medical information systems. Our proposed SVMI model offers a practical tool that can lead to great adoption by physicians and health care providers.

## Figures and Tables

**Table 1:** Analysis of the answers to the first question: Analyzing Figure 13a (non-contrast MDCT image of the patient’s endocranium), what is your opinion on whether there are changes in the brain, and if there are, what changes are possible?

**Neuroradiology/Radiology Specialists**	**n = 9**	**%**
Accurate diagnosis	6	66.7%
Performed characterization without a differential diagnosis	1	11.1%
Performed differential diagnosis including acute ischemic stroke	2	22.2%
**MDs in the Radiology Residency Training Program**	**n = 11**	**%**
No changes were observed	5	45.5%
Performed differential diagnosis excluding acute ischemic stroke	1	9.1%
Inadequate differential diagnosis	3	27.3%
Observed changes without characterization	1	9.1%
Inadequate characterization	1	9.1%

**Table 2:** Analysis of the given answers to the second question: Analyzing Figure 13b (SVMI of the non-contrast MDCT image of the endocranium of the same patient and the same region and cross-section), in your opinion, what is the possible diagnosis?

Neuroradiology/Radiology Specialists, and MDs in the Radiology Residency Training Program	n = 20	%
Accurate diagnosis	19	95.0%
Incorrect diagnosis	1	5.0%

**Table 3:** Analysis of the answers given to the third question: Please express position and confidence in a given opinion based on observation of a Figure 13b.

Yes, I Am	To Some Extent, I Am	I Have No Stance	To Some Extent, I’m Not	I’m Not
10 (50%)	7 (35%)	/	1 (5%)	2 (10%)

**Table 4:** Analysis of the given answers to the final question: By analyzing Figure 13b, does this smart visualization (SVMI) of the medical images of a patient’s brain contribute to the education of MDs in the radiology residency training program and undergraduate medical students?

It Can	To Some Extent It Can	I Have No Stance	To Some Extent It Cannot	It Cannot
12 (60%)	7 (35%)	/	/	1 (5%)

## Data Availability

The data presented in this study are available from the corresponding author upon reasonable request.
